# Contrasting Theories of Interaction in Epidemiology and Toxicology

**DOI:** 10.1289/ehp.1205889

**Published:** 2012-09-26

**Authors:** Gregory J. Howard, Thomas F. Webster

**Affiliations:** 1 Environmental Studies Department, Dickinson College, Carlisle, Pennsylvania, USA; 2 Environmental Health Department, Boston University School of Public Health, Boston, Massachusetts, USA

**Keywords:** antagonism, concentration addition, interaction, mixtures, synergy, TEF, toxic equivalency factor

## Abstract

Background: Epidemiologists and toxicologists face similar problems when assessing interactions between exposures, yet they approach the question very differently. The epidemiologic definition of “interaction” leads to the additivity of risk differences (RDA) as the fundamental criterion for causal inference about biological interactions. Toxicologists define “interaction” as departure from a model based on mode of action: concentration addition (CA; for similarly acting compounds) or independent action (IA; for compounds that act differently).

Objectives: We compared and contrasted theoretical frameworks for interaction in the two fields.

Methods: The same simple thought experiment has been used in both both epidemiology and toxicology to develop the definition of “noninteraction,” with nearly opposite interpretations. In epidemiology, the “sham combination” leads to a requirement that noninteractive dose–response curves be linear, whereas in toxicology, it results in the model of CA. We applied epidemiologic tools to mathematical models of concentration-additive combinations to evaluate their utility.

Results: RDA is equivalent to CA only for linear dose–response curves. Simple models demonstrate that concentration-additive combinations can result in strong synergy or antagonism in the epidemiologic framework at even the lowest exposure levels. For combinations acting through nonsimilar pathways, RDA approximates IA at low effect levels.

Conclusions: Epidemiologists have argued for a single logically consistent definition of interaction, but the toxicologic perspective would consider this approach less biologically informative than a comparison with CA or IA. We suggest methods for analysis of concentration-additive epidemiologic data. The two fields can learn a great deal about interaction from each other.

Environmental exposures often consist of exposure to multiple agents. A complete understanding of interactive effects would require both the ability to identify unexpected interactions in order to improve our biological understanding of mechanisms, and an ability to predict combination effects in order to improve risk assessment and public health decision making.

Both epidemiologists and toxicologists approach interaction assessment by defining a noninteractive model; departures from the model are then considered interactive (e.g., synergistic or antagonistic). The choice of model—the definition of “noninteraction”—is the critical first step because appropriate models will lead to more biologically informative conclusions regarding synergistic or antagonistic action. The definition of noninteraction in the two fields, however, proceeds very differently.

Epidemiologists have divided interactions into several “contexts,” referring to statistical, biological, public health, and individual interactions (Blot and Day 1979; Rothman et al. 1980). Most methods that epidemiologists and biostatisticians use to assess interaction are explicitly statistical in nature and should not, as these authors pointed out, be expected to provide information about biologic mechanism of action. For inference about causal mechanisms, epidemiologists have often relied on a single criterion, the additivity of risk differences, considering deviation from risk difference additivity (RDA) to be the single appropriate metric for examination of biological interaction (Ahlbom and Alfredsson 2005). By contrast, toxicologists (and pharmacologists) have developed several models for biological noninteraction, each of which is rooted in a simple assumption about mode of action.

Although the word “interaction” is used in both fields, these divergent approaches have led to dramatically different understandings of its meaning. Here we explore these differences and demonstrate that some combinations considered noninteractive by toxicologists are likely to meet the criteria for interaction in an epidemiologic analysis.

## Epidemiologic Analysis of Interaction

Epidemiologic theory of biological interaction is rooted in counterfactual models that describe all possible responses of individuals to different patterns of exposure. Both the counterfactual susceptibility types (CFST) model and the sufficient component causes (“causal pies”) model are deterministic descriptions of binary outcomes due to dichotomous exposures, and are intended to define the range of possible biological outcomes without reference to any specific mechanism (Rothman et al. 2008).

The CFST model describes all possible ways by which individuals of different counterfactual susceptibility types could react to a binary exposure (Greenland and Poole 1988). This model is usually considered deterministic, where each individual always responds according to their type. Given a single binary exposure *X* with a binary outcome, there are four possible types for the exposures *x* = 0 (unexposed) and *x* = 1 (exposed) (Table 1) (Greenland and Robins 1986). From this description of the possible individual responses, one can construct the response in the population as a whole. Taking *p*_1_ through *p*_4_ to represent the proportion of each type in the population, one calculates risks in the population by simply adding proportions. For example, the risk in the population when exposed to *X* is simply *p*_1_ + *p*_2_ (types 3 and 4 do not have the outcome and thus do not contribute to the risk).

**Table 1:** CFST model for a single exposure.

Type	x = 1	x = 0	Description
1		1		1		Doomed
2		1		0		X causal
3		0		1		X preventive
4		0		0		Immune
Outcomes are given as 0 or 1 for the exposed (x = 1) and unexposed (x = 0) scenarios (Greenland and Robins 1986).						

The model is easily extended to two exposures (Greenland and Poole 1988). Having listed all possible response types for exposures *X* and *Z* (Table 2), one may describe a noninteractive population by eliminating the interactive types from the model (i.e., setting the proportion of those types in the population to zero). The interactive types were identified by Miettinen (1982) and clarified by Greenland and Poole (1988); many correspond to intuitive notions of synergy or antagonism. In the words of Rothman et al. (2008), “The defining feature of these 10 interaction types is that we cannot say what the effect of *X* will be … unless we know that person’s value for *Z* ….” This definition depends on the interdependence of action of causal factors; indeed, some authors refer to noninteraction more specifically as “noninterdependence” (Greenland and Poole 1988), and some authors use the terms interchangeably (Greenland 1993).

**Table 2:** CFST model for two exposures.

Type	x = 1 z = 1	x = 0 z = 1	x = 1 z = 0	x = 0 z = 0	Description
1		1		1		1		1		Doomed
2a		1		1		1		0		X causal, Z causal, joint causation by X + Z
3a		1		1		0		1		
4		1		1		0		0		Z causal, X ineffective
5a		1		0		1		1		
6		1		0		1		0		X causal, Z ineffective
7a		1		0		0		1		X preventive, Z preventive, X + Z antagonizes
8a		1		0		0		0		X + Z causal
9a		0		1		1		1		X + Z preventive
10a		0		1		1		0		X causal, Z causal, X + Z antagonizes
11		0		1		0		1		X preventive, Z ineffective
12a		0		1		0		0		
13		0		0		1		1		Z preventive, X ineffective
14a		0		0		1		0		
15a		0		0		0		1		X preventive, Z preventive, joint prevention by X + Z
16		0		0		0		0		Immune
Outcomes are given as 0 or 1. aInterdependent types according to Greenland and Poole (1988); noninterdependent types are shaded. See also Miettinen (1982).										

Applying this definition, an individual of type 4 will always have the outcome when *z* = 1, regardless of the value of *X*; for this individual, *Z* is causal and *X* ineffective. This is a noninterdependent (i.e., epidemiologically noninteractive) type, because the effect of *Z* can be predicted without knowledge of the status of exposure to *X*. By contrast, an individual of type 8 responds only to the combined exposure *X* + *Z*. *X* and *Z* are thus interdependent in this type of individual: Without knowing the individual’s *Z* exposure, one cannot predict the result of an exposure to *X*. This corresponds to an intuitive definition of synergy, where both exposures are required to produce an effect.

Eliminating interdependent types leaves only types 1, 4, 6, 11, 13, and 16 in our population. Risks under the various exposure scenarios are designated as *R_XZ_* [e.g., *R*_10_ is the risk in a population exposed to *X* (*x* = 1) but not to *Z* (*z* = 0)]. By writing down risks in the four possible exposure combinations and rearranging them, one obtains a simple equation for noninterdependence (Rothman et al. 2008):

(*R*_11_ – *R*_00_) = (*R*_10_ – *R*_00_) + (*R*_01_ – *R*_00_). [1]

The risk difference due to the joint exposure is simply the sum of the risk differences due to the individual exposures. Because Equation 1 was derived using only the noninterdependent types, a departure must imply the presence of interdependent types. Thus, RDA (additivity of the risk differences) is a criterion for noninterdependence. It is a necessary but not sufficient criterion, however, because interdependent types may occur in a population in such a way as to satisfy the RDA equation (Rothman et al. 2008). Therefore, deviation from RDA indicates interaction, but satisfying RDA does not prove lack of interaction. (We assume, here and below that bias and confounding are absent.)

The RDA criterion derives from counterfactual models describing biological responses without depending on any specific mechanism. Therefore, deviation from RDA is seen as the fundamental criterion for biological interaction in epidemiology: “an unambiguous definition of biologic interaction” (Rothman 2002). Although derived from binary models, the RDA criterion is also used for continuous epidemiologic exposures, including cholesterol, hypertension, age, coffee consumption, smoking, and others; these continuous values are typically categorized before RDA is applied (e.g., Hallqvist et al. 1996).

In practice, many of the epidemiologic parameters available for assessing interaction—including Koopman’s “interaction contrast” (IC), Rothman’s S index, and Walker’s attributable fraction due to interaction *I*(*A* × *B*)—are derived from the RDA criterion (Koopman 1981; Rothman 1976; Walker 1981). Other authors have demonstrated how to use Cox and logistic regression to find departures from additivity on the risk difference scale, with the explicit goal of assessing biological interaction (e.g., Andersson et al. 2005).

## The Sham Combination in Epidemiology

It has long been recognized that shape of the dose–response curve (DRC) complicates interaction assessment when exposures are continuous (Greenland 1993). Rothman (1974) demonstrated an important implication of RDA for this situation in an interesting thought experiment. Consider the construction of a response from a series of noninteracting “distinct causes” made up of successive doses of the same agent (assume that the outcome describes the risk in a population). Starting with an initial dose *X* of 1 unit, producing a risk *R*_10_, add an equal second dose, *Z*. Because the agents and doses are identical, *R*_10_ = *R*_01_. With no background risk, the RDA equation yields

*R*_11_ = *R*_10_ + *R*_01_ = 2*R*_10_. [2]

The “doubly exposed,” those receiving 2 units of dose, have twice the risk of the singly exposed. For this to be true, regardless of choice of dose, requires that the dose–response relationship be linear: The linear DRC represents noninteractivity (noninterdependence) under the RDA definition. Any nonlinear DRC will exhibit interdependence (synergy or antagonism) and may, in fact, exhibit synergy in one dose range and antagonism in another. Intuitively, when the dose–response relationship is nonlinear, we require knowledge of the initial dose—the position along the DRC—to predict the additional effect of the subsequent dose. The sham combination is a thought experiment, not a method used by epidemiologists to examine the shapes DRCs. Nevertheless, these conclusions logically follow from the use of noninterdependence to derive RDA.

## Toxicologic Analysis of Interaction

Unlike epidemiologists, toxicologists’ ideas about interaction do not start with counterfactual models (i.e., describing responses of different types of individuals). Because modern toxicologists can control both the exposures and randomization of nearly identical subjects, they are typically little concerned with confounding or bias; from an epidemiologic point of view, they are essentially studying one type of individual. Although counterfactual models could be constructed for studies of diverse animal populations, they would require a large number of types to support the continuous exposures (and often continuous outcomes) of interest in toxicology. For example, some older toxicologic studies used a “quantal” model describing binary outcomes that occur when an individual’s tolerance for an exposure is exceeded (Finney 1971). This model is closely related to the CFST model because it deterministically predicts each individual’s outcome for any exposure condition once the individual’s threshold is known. (It could be expanded to include “doomed” individuals that develop disease with no exposure, or “immune” individuals that have an infinite threshold.) By analogy with the CFST model, each possible exposure threshold is a different susceptibility type; therefore, the continuous quantal model contains an infinite number of possible types. Instead of using counterfactual models, however, toxicologists begin the study of interaction with DRCs.

Like epidemiologists, toxicologists define “interaction” as a departure from a noninteraction criterion, usually called the “null model” in toxicology. Unlike epidemiologists, toxicologists use several different null models. Starting with a null model, and the DRCs of each agent given individually, it is possible to construct the expected (noninteractive) response to a combination exposure. This approach dates back to Bliss (1939), who defined “synergistic action” as any case in which “the effectiveness of the mixture cannot be assessed from that of the individual ingredients ….” For a toxicologist, a joint effect is considered noninteractive if it follows a simple biological expectation that can be predicted from the responses of the individual agents. (Null models are often said to describe “additive” effects; we avoid that term due to its potential for confusion.) These null models are also used in risk assessment to predict expected effects when more specific mechanistic details are not known [U.S. Environmental Protection Agency (EPA) 2000].

The simplest null model, commonly used by toxicologists by implication and often without justification, is effect summation:

*f*(*x,z*) = *f*(*x*,0) + *f*(0,*z*), [3]

where *f*(*x,z*) describes the joint response for exposure to agents *x* and *z*; *f*(*x*,0) and *f*(0,*z*) describe the DRC for each agent individually. Whether this definition includes a background response is not usually made explicit; for a more precise definition, we might subtract the background effect *f*(0,0) from each term:

*f*(*x,z*) – *f*(0,0) = *f*(*x*,0) – *f*(0,0) + *f*(0,*z*) – *f*(0,0). [4]

The epidemiologic RDA criterion (Equation 1) is a special case of Equation 4 when the exposures are dichotomous.

## The Sham Combination in Toxicology

Effect summation may be intuitive (many toxicologists use it implicitly and uncritically), but it is generally considered insufficient. The major toxicologic argument against it is the “sham combination,” a thought experiment essentially identical to the one by Rothman described above but interpreted very differently (Berenbaum 1989).

Consider a “combination” of two exposures, *A* and *B*, which actually consist of the same toxic agent. We take *f*(.) as describing the causal effect above the background. Effect summation (Equation 3) requires

*f*(*A,B*) = *f*(*A*,0) + *f*(0,*B*), [5]

but because *B* is identical to *A*, this is equivalent to

*f*(*A* + *B*) = *f*(*A*) + *f*(*B*). [6]

This last condition is met only if the DRC is linear in *A*. For example, if *A* = *B*, then *f*(*A* + *B*) = *f*(2*A*) = 2*f*(*A*).

**Figure 1:**
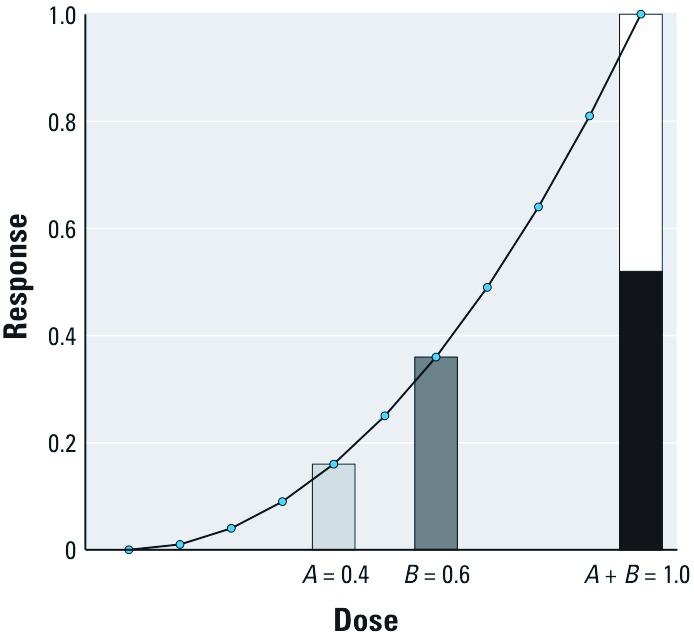
The “sham combination” of two identical agents, in doses *A* = 0.4 and *B* = 0.6, yields a larger response than the sum of the individual effects if the dose–response curve has increasing slope.

What happens if the DRC is not linear? Suppose it increases more rapidly than a linear response (Figure 1). With “sham” doses *A* = 0.4 units and *B* = 0.6 units of the same agent, the effect of the combination dose *f*(*A* + *B*) = *f*(0.4 + 0.6) = *f*(1.0) is much greater than the sum of the individual responses *f*(*A*) + *f*(*B*).

The toxicologic sham combination yields the same result we saw in Rothman’s thought experiment: *f*(*A*,*B*) = *f*(*A*,0) + *f*(0,*B*) only if *f*(.) is linear; that is, effect summation holds only for linear dose response. Toxicologists do not consider nonlinearity to have special biological significance, but consider the DRC of an agent to be a property of that agent (more precisely, a property of the agent working on a particular tissue or system). From a mechanistic point of view, many nonlinear DRCs can be modeled by simple biological mechanisms (e.g., receptor filling) that toxicologists do not consider “interactive” in any important biological sense. Indeed, Kortenkamp and Altenburger (1998) stated that “the conclusion that an agent interacts with itself in a synergistic way is absurd.” (There may be interesting exceptions, however. For example, the structural change in hemoglobin that results from allosteric binding of oxygen increases the availability of other binding sites to subsequent oxygen molecules. This is often referred to as “cooperativity.”)

If one considers different doses of an agent as not synergizing with (or antagonizing) each other, and the nonlinear DRC is therefore noninteractive, then the sham combination itself must be the noninteractive condition. This is the basis for the null model that Bliss (1939) called simple similar action, which is now called concentration addition (CA) or dose addition.

The CA model is most easily derived for the case of joint exposure to two agents, *A* and *B*, which are not identical but have parallel DRCs differing only by a factor of potency (i.e., only in the amount of the agent required to produce the same effect). In that case, *A* can be considered a dilution of *B* by some factor γ, such that *f_A_*(*A*) = *f_B_*(γ*A*). By the same logic, we can substitute for the joint effect *f_AB_*(*A,B*) = *f_B_*(γ*A* + *B*). Consider a specific response level *E*, which is caused by either doses *A_E_* or *B_E_* when given independently [i.e., *E* = *f_A_*(*A_E_*) = *f_B_*(*B_E_*)]. Any combination dose (*A,B*) will also cause *E* if it satisfies

. [7]

Equation 7 defines the null model of concentration addition. (The noninteractive sham combination is the special case where *A* is identical to *B*.) Because this derivation assumes that exposures *A* and *B* can be substituted for one another in proportion to their potencies, CA is usually considered appropriate for agents that act via a “similar” mechanism (U.S. EPA 2000), although there has been considerable discussion as to the meaning of “similar” (Borgert et al. 2004). In assuming that *A* was a dilution of *B*, this derivation followed the toxic equivalency factor (TEF) model, a special case of CA where the relative potency γ is constant for all effect levels. This is the best-known implementation of CA, commonly used for assessing and predicting noninteractive effects of combinations of dioxin-like agents (Van den Berg et al. 2006). However, the assumption of a constant value of γ = *B_E_*/*A_E_* is not a requirement of the CA definition; more generally, γ can vary with effect level *E* if *A* and *B* have nonparallel DRCs (Howard and Webster 2009).

CA has rarely been mentioned by epidemiologists as a method for analyzing interaction (Cornfield 1975; Thomas and Whittemore 1988), yet it is widely used by toxicologists, particularly in the TEF form. Just as we expressed the total effect of the joint exposure (*A,B*) in terms of an isoeffective dose of *B* given by γ*A* + *B*, the TEF method allows toxicologists to express a mixture of similarly acting polychlorinated dioxins, polychlorinated dibenzofurans, and polychlorinated biphenyls by a single equipotent dose.

Another important null model used by toxicologists is Bliss’s “independent joint action” (IA) (Bliss 1939). This model describes causal action in stochastic terms, where the joint outcome is the probabilistic sum,

P(*A* + *B*) = P(*A*) + P(*B*) – P(*A*)P(*B*). [8]

IA depends on the statistical independence of the two exposures; the same model has been discussed by epidemiologists (Rothman 1974; Weinberg 1986). In the case of low risks, when the product term may be ignored, it is approximated by RDA (Rothman 1974). Similarly, effect summation is sometimes used by toxicologists and risk assessors as a low-risk approximation to IA (U.S. EPA 2000).

These three null models—IA, CA, and effect summation—are those most commonly used in toxicology. For toxicologists, CA and IA have firm biological foundations based on assumptions about mode of action; effect summation does not. In epidemiology, there is still discussion about the appropriate measure for interaction assessment, including the choice between the additive (risk difference) or multiplicative (risk ratio) scales (Weinberg 2012). CA, however, occurs on neither of these scales. Instead of adding or multiplying the effects of individual agents, CA involves addition of weighted exposures (i.e., isoeffective doses). CA’s inherent dependence on the shape of the DRC means that a straightforward mathematical combination (e.g., addition or multiplication) of the outcomes (i.e., the joint effects or risks) will not be an adequate description of joint action for a concentration-additive combination unless the dose response is linear.

## CA and RDA

We have seen that RDA is a special case of the toxicologic model of effect summation. Here we examine its usefulness in evaluating concentration-additive exposures.

Counterfactual type 2 is a particularly interesting example: This is the only type in which each exposure is effective individually and the joint exposure is also effective. From the epidemiologic perspective, it is interdependent (Greenland and Poole 1988) because for the binary outcome of the CFST model, each exposure causes an effect only if the other is absent. Although this conclusion is logical, toxicologists would not consider it informative in the intended biological sense.

For example, one of the most common DRCs in toxicology is the Hill function,

^,^ [9]

**Figure 2:**
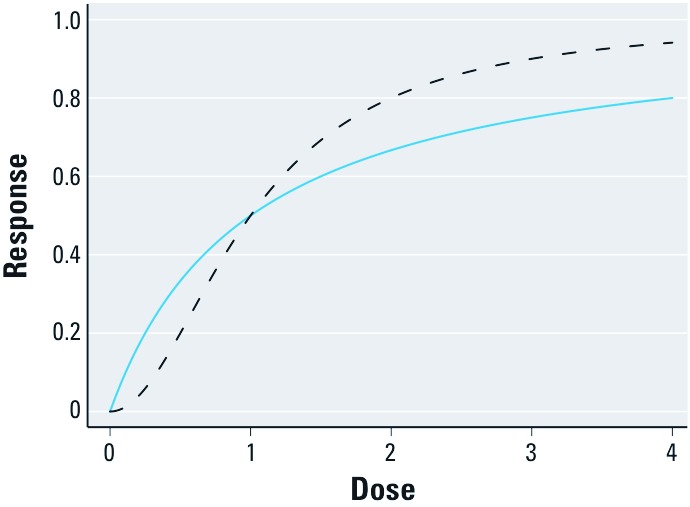
Hill functions with slope parameters *n* = 1 (blue line) and *n* = 2 (dashed line). In each case, *K* = 1.

where *K_A_* is the dose producing a half-maximal effect—a measure of potency—and *n* is a slope parameter. When *n* = 1, the DRC for a single agent increases less rapidly than linear (Figure 2), and a sham substitution would be considered antagonistic using effect summation (or RDA).

Consider a TEF model in which *B* has a relative potency γ compared with *A*; that is, *f_A_*(*A*) = *f_B_*(γ *A*). The joint effect for two (*n* = 1) agents is given by

. _[10]_

**Figure 3:**
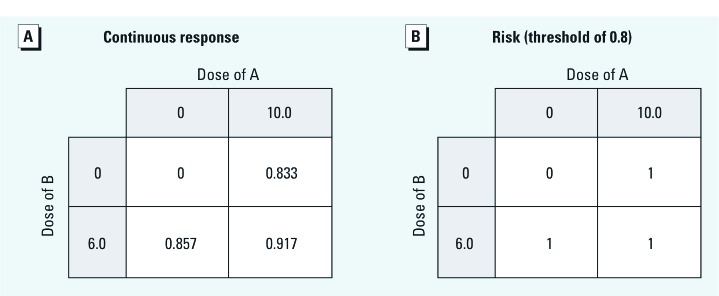
Responses to the *n* = 1 TEF joint exposure.

Any given effect level between 0 and 1 can be achieved (or any tolerance exceeded) using *A* alone, *B* alone, or a combination of *A* and *B*. (The maximum possible effect of 1 is reached in the limit of large dose.) If *K_A_* = 2 and *K_B_* = 1, *A* is half as potent as *B*, and γ = 0.5; by setting the exposure for each agent to a large value compared with its own *K*, we can calculate the responses to two dichotomous doses (Figure 3A).

Suppose that this thought experiment takes place in a population of identical individuals who, when subjected to this combination exposure, will suffer a binary health outcome above a response threshold of 0.8. The risks under these dichotomous exposure conditions are given in Figure 3B; each individual responds according to the type 2 pattern. This is an interdependent type because the outcome of exposure to *A* differs in strata of *B*. [Alternatively, consider a population of identical responders for whom Equation 10 gives the probability of a binary outcome; because the individuals are identical, the responses in Figure 3A directly describe the population-level risks, which we may then use to test for epidemiologic interaction. Application of the interaction contrast or Rothman’s S (synergy) index (Rothman 1976) to these risks yields IC = –0.774 and S = 0.54, both clearly indicating interaction relative to RDA.] Yet from a toxicologic perspective, the system is concentration additive and noninteractive. Furthermore, Equation 10 is consistent with saturation of a receptor system, not considered a case of biological interaction by toxicologists.

Now consider counterfactual type 8, where the outcome occurs only with exposure to both agents. From the epidemiologic perspective, this interdependent type is intuitively synergistic. We can, however, produce results consistent with this situation using a CA model. Consider a Hill function with *n* = 2 (Figure 2); many estrogenic agents have a value of *n* between 2 and 3 (Silva et al. 2002). Applying the TEF model, the joint effect is

[11]

**Figure 4:**
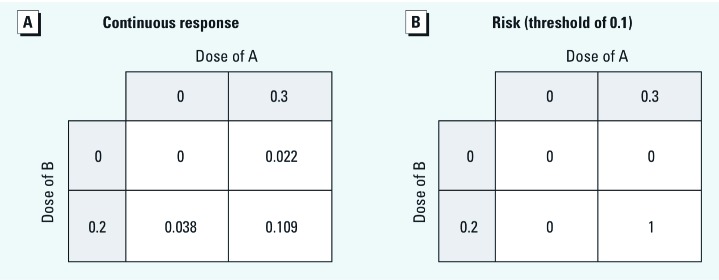
Responses to the *n* = 2 TEF joint exposure.

Again taking *K_A_* = 2 and *K_B_* = 1 and choosing dichotomous doses for the “exposed” that are very low compared with *K_A_* and *K_B_*, we obtain Figure 4A. Suppose that this experiment takes place in identical individuals. For a binary outcome defined by a threshold of 0.1, the risks are 1 in the doubly exposed and 0 elsewhere (Figure 4B), following the pattern of type 8 (synergistic) individuals. (For the probabilistic interpretation of Figure 4A, application of IC or the S index shows strong synergy with respect to RDA: IC = 0.049 and S = 1.8.) The choice of a different, much lower threshold in determining Figure 4B would produce results that appear characteristic of type 2 individuals. This change in results occurs because Figure 2 rises steeply at low doses and flattens at high doses. From a toxicologic point of view, there is nothing remarkable about such a DRC or interactive about these results: The system is concentration additive.

Finally, we consider experimental evidence for such systems. Silva et al. (2002) tested a mixture of eight estrogenic agents in a yeast culture assay, and found that the results followed the CA model. Importantly, each of these agents was exposed below its no observed effect concentration or EC_01_ (the concentration producing 1% effect)—in either case, a dose producing a very low effect—but the mixture produced a strong effect, larger by a factor of 20 than that predicted by effect summation. Using the criterion of effect summation, this is a clear case of synergy. However, the results are not interactive under CA because the CA model accurately predicts the joint effect from the individual components. Because each agent acts as a dilute form of estrogen, the low doses combine to cause a single effect through their common pathway.

## Discussion

RDA derives from counterfactual models of susceptibility types. The underlying definition used to eliminate interactive types is that of interdependence. Basing interaction on this underlying idea seems intuitive. In individuals of identical CFST type, if the effect of *X* is the same regardless of any simultaneous exposure to *Z*, then Z evidently has no effect on *X*; that is, there is no dependence of the effect of *X* on the effect of *Z* (or vice versa).

Application of the RDA criterion to the sham substitution leads to the conclusion that a compound can synergize with or antagonize itself, or both, depending on the shape of the response and the specific doses evaluated. Under the epidemiologic definition, a nonlinear dose response must be seen as interactive, a conclusion toxicologists do not consider biologically insightful. Following Bliss (1939), toxicologists instead define “interaction” as a synergistic or antagonistic departure from an expected joint effect. Risk assessors use the same approach, reserving “interaction” for departures from a model based on the action of individual components (U.S. EPA 2000). This approach construes the DRC itself—and thus the sham combination—as noninteractive. For similarly-acting compounds, the result is the CA null model.

The epidemiologic and toxicologic perspectives rely on different definitions of interaction. Neither definition can be said to be true or false; the question is whether they lead to useful results. From the toxicologic perspective, the epidemiologic RDA criterion may supply little biologically useful information. Using simple biological models, we have shown that results consistent with two counterfactual susceptibility types considered by epidemiologists to be interdependent—type 2 and type 8—may be noninteractive from the toxicologic perspective. Concentration-additive exposures, like those in our examples, must always be interdependent because each agent acts by contributing to a single pathway. Rather than using noninterdependence, the toxicologic approach defines noninteractivity using null models based on general modes of action. Deviation from the null model then implies something unexpected about the underlying biology.

Epidemiology has the advantage of a single definition for judging interaction, which is rigorously and logically applied. There are three definitions for “noninteraction” in general use in toxicology, with one, effect summation, considered inappropriate by mixtures toxicologists. The other two—CA and IA—are used as null models for compounds with similar or different modes of action, respectively. This raises the question of what toxicologists mean by “similar,” a question that is still debated. Despite this important issue, there has not been a proliferation of toxicologic null models, each with its corresponding type of synergy or antagonism. Although more sophisticated models have sometimes been proposed (e.g., Rider and LeBlanc 2005), toxicologists and risk assessors have generally restricted themselves to the models (CA and IA) originally suggested by Bliss (U.S. EPA 2000). Indeed, toxicology may be approaching a point suggested by epidemiologists almost 30 years ago:

Such classification [of biological models for interaction] could be a useful shorthand to describe categories of mechanisms, but only if such categories were widely and explicitly agreed upon in the scientific community. (Rothman et al. 1980)

Which approach for assessing interaction yields the most insight? Or will combinations of the epidemiologic and toxicologic approaches work better? One approach to answering these questions is to apply both sets of methods to appropriate epidemiologic (or toxicologic) datasets. A necessary foundation for comparing results or sharing ideas across the two fields is an understanding of the terminology and underlying concepts and methods of each. Practical questions in each field must be taken into account. For example, assessing interaction can be problematic in many real-world epidemiologic studies given the primary concern with bias and confounding. Toxicology’s narrow focus on identical individuals limits its applicability in real-world situations; epidemiology’s emphasis on counterfactual modeling might help toxicologists develop better methods for situations when all individuals are not identical. For example, if a population consists of two types of individuals who respond with different sensitivities to two chemical exposures, it is possible to show that risks in the population as a whole need not be concentration additive, even if they are for each type of individual. Collaborative investigation of models like these may provide new insights for both fields.

For epidemiologists who wish to apply toxicologic methods for examining interaction, we provide these thoughts. When exposures operate through nonsimilar pathways, toxicologists consider IA the best default model. If analysis is limited to low effect levels (when the product term in Equation 8 is negligible), or in the rare case of linear response, the toxicologic (IA) and epidemiologic (RDA) approaches reach the same conclusion. Current epidemiologic methods for examining interaction can then be used with little or no modification. For exposures operating through similar pathways, however, this is not the case. As we have seen, the departure of concentration additive exposures from RDA may be substantial even at the lowest doses. Fortunately, some of the approaches discussed below are already used by epidemiologists to construct exposure measures or analyze data, although not for examining interaction.

For exposures thought to act by similar mechanisms and whose individual actions can be well characterized, a CA model can be used to predict the joint effects of combinations from mathematical functions describing the individual DRCs (e.g., Howard and Webster 2009). One can then construct noninteractive response surfaces for comparison with the data, testing for mode of action and for the presence of interactive effects (e.g., Howard et al. 2010).

More simply, similarly acting exposures can be grouped into a single equipotent exposure using a TEF-like model (or a generalized CA model). For dioxin-like agents, this is common practice (Van den Berg et al. 2006). Such a model is likely to be a productive route for examining exposure to estrogens and xenoestrogens, for example, or androgens and antiandrogens. More research is needed to generate the data necessary for this approach and to test its applicability.

A third approach might employ a simple biological assay to estimate the combined activity (e.g., dioxin-like or estrogenic) of a complex mixture (e.g., human serum). The assay result could then be used as the exposure measure for the mixture. A combination of analytical chemistry and toxicologic analysis could then be used to determine the contribution of individual components and whether they explain the activity of the mixture.

In many cases, little detailed dose–response information is available for human populations. Here one might use the “method of isoboles”: When the concentration of one agent is plotted against the concentration of the other, curves of constant joint effect (isoboles, or contours of the response surface) must be negatively sloped straight lines if the exposures are concentration additive. This simple visual analysis is amenable to use even with very small numbers of data points (Berenbaum 1989). Isobolographic analysis and related methods for response surface analysis (Greco et al. 1995) may be useful when analyzing epidemiologic data, which is typically spaced irregularly in the concentration-concentration plane.

Interaction, and the terminology used to describe it, has long been a source of confusion and debate in both epidemiology and toxicology (Ahlbom and Alfredsson 2005; Könemann and Pieters 1996). We strongly believe that increased discussion and collaboration between the two fields will increase our understanding of interaction.
